# Theories used to develop or evaluate social prescribing in studies: a scoping review

**DOI:** 10.1186/s12913-024-10563-6

**Published:** 2024-01-26

**Authors:** Sinah Evers, Kerryn Husk, Hendrik Napierala, Lydia Wendt, Ansgar Gerhardus

**Affiliations:** 1 Department of Health Services Research, Institute of Public Health and Nursing Research, University of Bremen, Grazer Straße 4, Bremen, 28359 Germany; 2 Health Sciences Bremen, University of Bremen, Bremen, Germany; 3 NIHR ARC South West Peninsula (PenARC), Faculty of Health, University of Plymouth, Plymouth, UK; 4 Charité – Universitätsmedizin Berlin, corporate member of Freie Universität Berlin and Humboldt-Universität Zu Berlin, Institute of General Practice and Family Medicine, Berlin, Germany; 5 School of Social Sciences, City University of Applied Sciences, Bremen, Germany; 6 Research Cluster ‘Healthy City Bremen’, Bremen, Germany

**Keywords:** Social prescribing, Health care research, Primary care, Social problems, Community, Theories

## Abstract

**Objective:**

This scoping review aims to provide an overview of how theories were used in the development or evaluation of social prescribing (SP) intervention studies.

**Background:**

SP describes a patient pathway where general practitioners (GPs) connect patients with community activities through referrals to link workers. This review seeks to understand the explanations provided for the outcomes and implementation process of SP.

**Inclusion criteria:**

Studies using a defined theory to develop or evaluate a specific SP intervention in primary care and the community sector.

**Methods:**

This scoping review was conducted in accordance with JBI methodology. The following databases were searched on 8^th^ of July 2022: PubMed, ASSIA, Cochrane, Cinahl, PsycINFO, Social Care Online, Sociological Abstracts, Scopus, and Web of Science. The search only considered English language texts. Additional literature was identified by searching relevant web pages and by contacting experts. The selection of sources and the data extraction was done by two reviewers independently.

**Results:**

The search resulted in 4240 reports, of which 18 were included in the scoping review. Of these, 16 were conducted in the UK, one in Canada and one in Australia. The majority of reports employed a qualitative approach (11/18). Three were study protocols. 11 distinct theories were applied to explain outcomes (4 theories), differences in outcomes (3 theories), and the implementation of the intervention (4 theories). In terms of practical application, the identified theories were predominantly used to explain and understand qualitative findings. Only one theory was used to define variables for hypothesis testing. All theories were used for the evaluation and none for the development of SP.

**Conclusion:**

The theories influenced which outcomes the evaluation assessed, which causal pathway was expected to generate these outcomes, and which methodological approaches were used. All three groups of theories that were identified focus on relevant aspects of SP: fostering positive patient/community outcomes, addressing inequalities by considering the context of someone’s individual circumstances, and successfully implementing SP by collaboratively working across professions and institutional boundaries. Additional insight is required regarding the optimal use of theories in practical applications.

**Supplementary Information:**

The online version contains supplementary material available at 10.1186/s12913-024-10563-6.

## Background

Social prescribing (SP) is ‘a way of linking patients in primary care with sources of support within the community to help improve their health and well-being’ [1]. It typically involves a link worker [2]. For example, a general practitioner (GP) might identify the impact of loneliness or financial difficulties on a patient’s health but lacks the necessary resources or time to address these issues effectively. SP enables the GP to send the patient to a link worker who can dedicate ample time for a comprehensive ‘what matters to you’ type conversation and help them in assessing their own needs as well as their resources. The patient is sometimes called participant or client at this point. Link workers subsequently recommend activities or support, usually within the categories of physical activity, nature-based, arts/culture, or advice [3]. Therefore, the SP patient pathway includes an initial referral, a linking function, and an activity [2].

There is a growing interest in SP in many countries [4]. From 2016, the United Kingdom has formalised social prescribing in central health policy and equipped it with significant funding. One aim of their national health Long Term Plan is to make SP accessible for every patient and GP by employing several thousand link workers [5].

Nevertheless, the definitions, activities, and outcome parameters of SP differ [6]. Systematic reviews usually concern well-being and health [1, 6–8], but further parameters include loneliness, social isolation, connectedness [6], health-related behaviour [7, 8], self-concepts, social contacts, day-to-day functioning [8], usage of health services [1], and economic measures [7]. Thereby, SP is not only aimed at addressing the individual level, but also the community and system level [6].

Varying aims, outcome parameters, and intervention designs result in difficulties in assessing the impact of SP and generate mixed results regarding effectiveness [9, 10]. Our assumption is that the different interventions and evaluations are explicitly or implicitly based on different theories or concepts. The aim of this review is therefore to identify theories applied for the implementation and evaluation of SP. These theories might explain the different ways how SP is expected to function as well as the variety of outcomes proclaimed to be achieved. Being aware of existing theories and their utilisation can strengthen the theoretical foundation of the intervention and provide necessary information for implementers and researchers. Establishing a strong theoretical foundation for interventions can improve their effectiveness by offering a thorough understanding of the underlying mechanisms and expected functionality [11]. The specific aims of this scoping review were threefold: (1) to identify studies that described how they used one or several theories for developing or evaluating a SP intervention, (2) to provide a comprehensive overview of the studies and theories, including relevant aspects like study type and how the theory was used, and (3) to categorise the theories and describe them, drawing a connection to the studies they were used in, thereby offering insights into the interconnections between these aspects.

A theory is defined for our purposes as a ‘set of analytical principles or statements designed to structure our observation, understanding, and explanation of the world’ [12], explains relationships between variables, and allows for predictions [12]. To our knowledge, there is no overview of theories for social prescribing yet.

### Review questions

1. Which theories are described in studies to develop or evaluate social prescribing?
2. What components or mechanisms do the theories include?
3. How did the studies use the theories?

## Methods

### Guidelines and protocol

This scoping review was conducted in accordance with the JBI methodology for scoping reviews [13] and the findings are reported in accordance with the PRISMA-ScR [14]. The protocol was prospectively deposited in Open Science Framework [15].

### Inclusion criteria

The intervention in the study had to include (1) a patient whose well-being or health is supposed to be improved, (2) a GP who can refer the patient (3) to a link worker who offers support and can refer the person (4) to further non-medical sources of support, often within the community. Reports were included independently of what population was addressed by the SP intervention but interventions must have been conducted in the primary care sector and in the community. There was no exclusion based on country or region.

Additionally, to be included, there needed to be a description of how the theory was applied. For example, a report was excluded if it simply stated that the underlying principle of SP can be explained by a specific theory. The definition we employed asserts that a theory is typically both explanatory and descriptive, encompassing variable definitions, establishing relationships among variables, and formulating precise predictions [12].

We considered all qualitative, quantitative and mixed methods studies that included an intervention. We also included protocols because we were interested in the description of how the theory was used and not necessarily in the results of the study. Reviews and opinion papers were not considered for inclusion. Reports not in the English language were excluded as well.

### Search strategy

The search strategy was developed with the support of a research librarian and combined a systematic approach of database searching with web-searching and contacting experts.

To identify relevant terms for the database search, a pilot search was conducted in PubMed using terms that are commonly associated with SP or have been identified in systematic reviews [1, 6, 7, 16–18]. In order to systematically search for theories, the following terms are recommended to be added to a search string: concept*, framework*, model*, and theor* [19]. However, during a preliminary search, we observed that this approach resulted in the omission of several relevant reports. Consequently, we opted not to include these terms in our final search strategy.

The pilot search in PubMed led to 740 results on July 19, 2022: ‘social prescri*’[Tiab] OR ‘social referral*’[Tiab] OR ‘community referral*’[Tiab] OR ‘community prescri*’[Tiab] OR ‘community-based prescri*’[Tiab] OR ‘community-based referral*’[Tiab] OR ‘link worker*’[Tiab] OR ‘community connector*’[Tiab].

The individual search strategy for each database as well as the number of results are in the Supplementary file 1, Table 2. Searches were conducted in PubMed, ASSIA, Cochrane, Cinahl, PsycINFO, Social Care Online, Sociological Abstracts, Scopus, and Web of Science.

Beyond relying on published peer-reviewed literature documented in formal databases we also searched websites of key organisations and contacted experts in the field via Twitter and email. Details of the additional search are provided in the Supplementary file 2.

### Selection of sources

Following the database search, all identified citations were collated and uploaded into EndNote 20 (Clarivate Analytics, PA, USA). Duplicates were removed [20]. Thereafter, all sources were imported into Rayyan for screening [21]. After conducting a pilot test, two independent reviewers screened the titles and abstracts to assess their compliance with the inclusion criteria for the review. Only the criterion ‘use of theory’ was not applied, as that could only be evaluated in the full-text. Potentially relevant sources were retrieved in full. The full texts of selected citations were assessed in detail against the inclusion criteria by two reviewers independently. At the full-text screening phase, the reasons for exclusion were recorded. Any disagreements between the reviewers at each stage of the selection process were resolved through discussion and if no consensus was reached, an additional reviewer was consulted.

### Data extraction

Data was charted using a tool designed by all authors. A preliminary tool was tested on five reports, with each author using it on one report. After the pre-test phase, two researchers independently extracted data from the included papers. One author (SE) extracted information from each report, while additionally the other authors divided all reports among themselves. Disagreements were resolved through discussion or consultation with an additional reviewer. Changes to the extraction tool compared to the protocol were made to align extracted information with the research questions (Supplementary file 3: Data extraction instrument). Study characteristics and the use of the theory were extracted from the full-text. They included author, year, title, country, publication type, methods, and theory, as well as how the theory was used (for example, as basis for a topic guide for interviews to evaluate SP). Information on the theories was collected from documents referenced in the respective reports.

### Synthesis approach

Data on study characteristics was tabulated and presented as characteristics of included studies. The theories used, and how they were used, was tabulated and narratively summarised.

## Results

### Search results

The combined search provided 4240 results, of which 3972 were from the database search and 268 from the additional search. Of the 3972 results the database search provided 2350 were duplicates. Two researchers independently screened the remaining 1622 titles/abstracts, resulting in the exclusion of 1480 reports. From the remaining 142 potentially relevant reports, 141 full texts were obtained (one was unavailable). Among these, 125 were excluded based on the eligibility criteria, leaving 16 reports to be included.

**Fig. 1:**
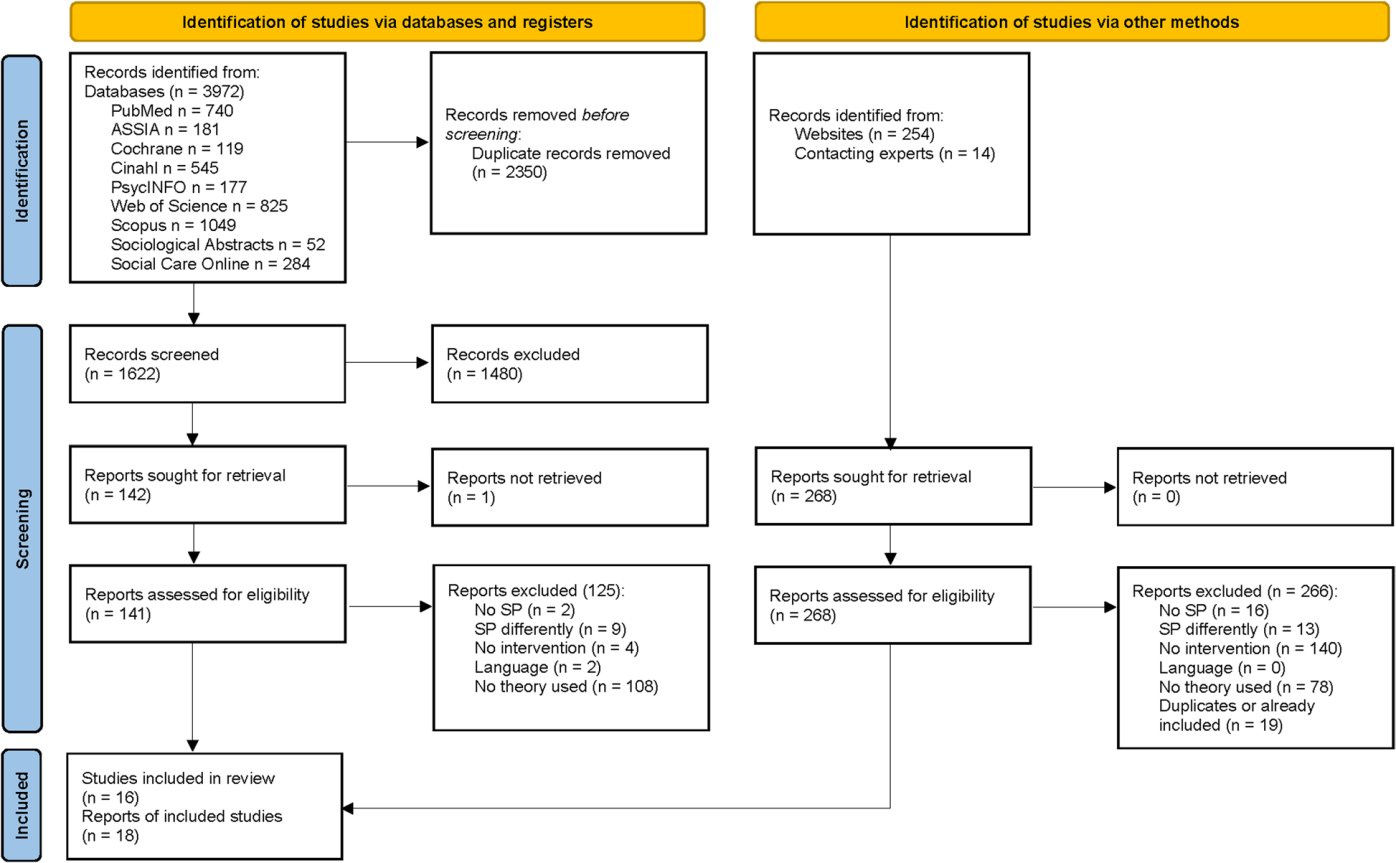
PRISMA flow diagram

Additionally, 254 reports were identified through website searches and 14 through a Twitter call and by contacting 29 experts by email (in October 2022). The 268 reports were retrieved and 249 were excluded based on the inclusion criteria. Of the remaining 19 reports, some were duplicates, for example if several experts named the same report, and some were already included in the results of the database search. After all duplicates were removed, two reports were added from the additional search to the included results, leading to a final number of 18 included reports. Because three reports belong to the same study, 18 reports represent 16 different studies. The Prisma flow diagram (Fig. 1) shows this in detail.

### Inclusion of sources

The included reports were published between 2014 and 2022. Sixteen were conducted in the UK, one in Canada and one in Australia. Seventeen were peer-reviewed journal articles and one was a grey literature evaluation. The reported study designs were qualitative (11), mixed methods (5), and quantitative (2). Three of these were protocols. Detailed information on each study can be found in Table 1.

**Table 1:** Study characteristics

No	Author(s)	Year	Title	Country	Publication type	Methods	Theory
1	Baker & Irving	2016	Co-producing Approaches to the Management of Dementia through Social Prescribing	UK	Journal article	Qualitative: interviews & focus groups with health commissioners, GPs, community health nurses, sheltered accommodation managers, people with dementia & family members	Boundary-spanning
2	Bhatti, Rayner, Pinto, Mulligan & Cole	2021	Using self-determination theory to understand the social prescribing process: a qualitative study	Canada	Journal article	Qualitative: focus groups & interviews with patients	Self-Determination theory (SDT)
3	Blickem, Kennedy, Jariwala, Morris, Bowen, Vassilev, Brooks, Blakeman & Rogers	2014	Aligning everyday life priorities with people’s self-management support networks: an exploration of the work and implementation of a needs-led telephone support system	UK	Journal article	Qualitative: interviews, focus group & observations with participants & support workers	Normalisation Process Theory (NPT)
4^a^	Chng, Hawkins, Fitzpatrick, O’Donnell, Mackenzie, Wyke & Mercer	2021	Implementing social prescribing in primary care in areas of high socioeconomic deprivation: process evaluation of the ‘Deep End’ community Links Worker Programme	UK	Journal article	Qualitative: focus groups, email surveys & interviews with GPs, community link practitioners, practice manager, practice nurses & community organisational workers	Normalisation Process Theory (NPT)
5	Dayson	2017	Evaluating social innovations and their contribution to social value: the benefits of a ‘blended value’ approach	UK	Journal article	Mixed methods: hospital episodes data; a pre/post wellbeing questionnaire with patients; interviews with patients, their carers, commissioners & providers	Social Innovation
6	Dingle, Sharman, Hayes, Chua, Baker, Haslam, Jetten, Haslam, Cruwys & McNamara	2022	A controlled evaluation of the effect of social prescribing programs on loneliness for adults in Queensland, Australia (protocol)	Australia	Journal article	Protocol for a controlled evaluation: Non-randomised controlled design with a SP condition & a primary care treatment as usual condition	Social Cure
7	Fixsen, Seers, Polley & Robins	2020	Applying critical systems thinking to social prescribing: a relational model of stakeholder “buy-in”	UK	Journal article	Qualitative: interviews, planning meetings & discussions with key staff, other stakeholders & service users	Critical Systems Thinking (CST)
8	Gibson, Pollard & Moffatt	2021	Social prescribing and classed inequality: A journey of upward health mobility?	UK	Journal article	Qualitative: ethnographic case study: interviews with participants & family members; participant observation	Bourdieusian approaches to class
9	Gibson, Moffatt & Pollard	2022	‘He called me out of the blue’: An ethnographic exploration of contrasting temporalities in a social prescribing intervention	UK	Journal article	Qualitative: ethnography, 200 h spent with participants and/or their families	Synchronicity, time
10	Halder, Wakefield, Bowe, Kellezi, Mair, McNamara, Wilson & Stevenson	2021	Evaluation and exploration of a social prescribing initiative: Study protocol	UK	Journal article	Protocol for mixed methods: survey; interviews with service-users and service-providers	Social Cure
11	Hanlon, Gray, Chng & Mercer	2021	Does Self-Determination Theory help explain the impact of social prescribing? A qualitative analysis of patients’ experiences of the Glasgow ‘Deep-End’ Community Links Worker Intervention	UK	Journal article	Qualitative: interviews with patients	Self-Determination Theory (SDT)
12	Kellezi, Wakefield, Stevenson, McNamara, Mair, Bowe, Wilson & Halder	2019	The social cure of social prescribing: a mixed-methods study on the benefits of social connectedness on quality and effectiveness of care provision	UK	Journal article	Mixed methods: interviews with GPs, Health Coaches, Link Workers & patients; longitudinal survey with patients	Social Cure
13^a^	Mercer, Fritzpatrick, Grant, Chng, O’Donnell, Mackenzie, McConnachie, Bakhshi & Wyke	2017	The Glasgow ‘Deep End’ Links Worker Study Protocol: a quasi-experimental evaluation of a social prescribing intervention for patients with complex needs in areas of high socioeconomic deprivation	UK	Journal article	Protocol for mixed methods: interviews with patients, managers of community organisations, Link Workers, GPs, reception staff & practice managers; Data mapped for interventions & controls	Self-Determination Theory (SDT); Candidacy theory; Normalisation Process Theory (NPT)
14^a^	Mercer, Wyke, Fitzpatrick, McConnachie, O’Donnell, Mackenzie, Bakhshi, Chng, Grant & McLeod	2017	Evaluation of the Glasgow ‘Deep End’ Links Worker Programme	UK	Grey literature evaluation	Mixed methods: focus groups; email survey; interviews with practice staff and patients	Self-Determination Theory (SDT); Candidacy theory; Normalisation Process Theory (NPT)
15	Wakefield, Kellezi, Stevenson, McNamara, Bowe, Wilson, Halder & Mair	2022	Social Prescribing as ‘Social Cure’: A longitudinal study of the health benefits of social connectedness within a Social Prescribing pathway	UK	Journal article	Quantitative: longitudinal survey with participants	Social Cure
16	White, Cornish & Kerr	2017	Front-line perspectives on ‘joined-up’ working relationships: a qualitative study of social prescribing in the west of Scotland	UK	Journal article	Qualitative: interviews with prescribers and providers (third sector organisations)	Social Capital
17	Whitelaw, Thirlwall, Morrison, Osborne, Tattum & Walker	2017	Developing and implementing a social prescribing initiative in primary care: insights into the possibility of normalisation and sustainability from a UK case study	UK	Journal article	Qualitative: interviews with project steering group, wider primary care team & various community groups	Normalisation Process Theory (NPT)
18	Wood, Ohlsen, Fenton, Connell & Weich	2021	Social prescribing for people with complex needs: a realist evaluation	UK	Journal article	Qualitative: interviews with participants, staff & referrer (SP community anchor organisation)	Salutogenesis

^a^Several reports for the same study: one protocol and two publications with results

### Review findings

#### Theories and their use in studies

**Fig. 2:**
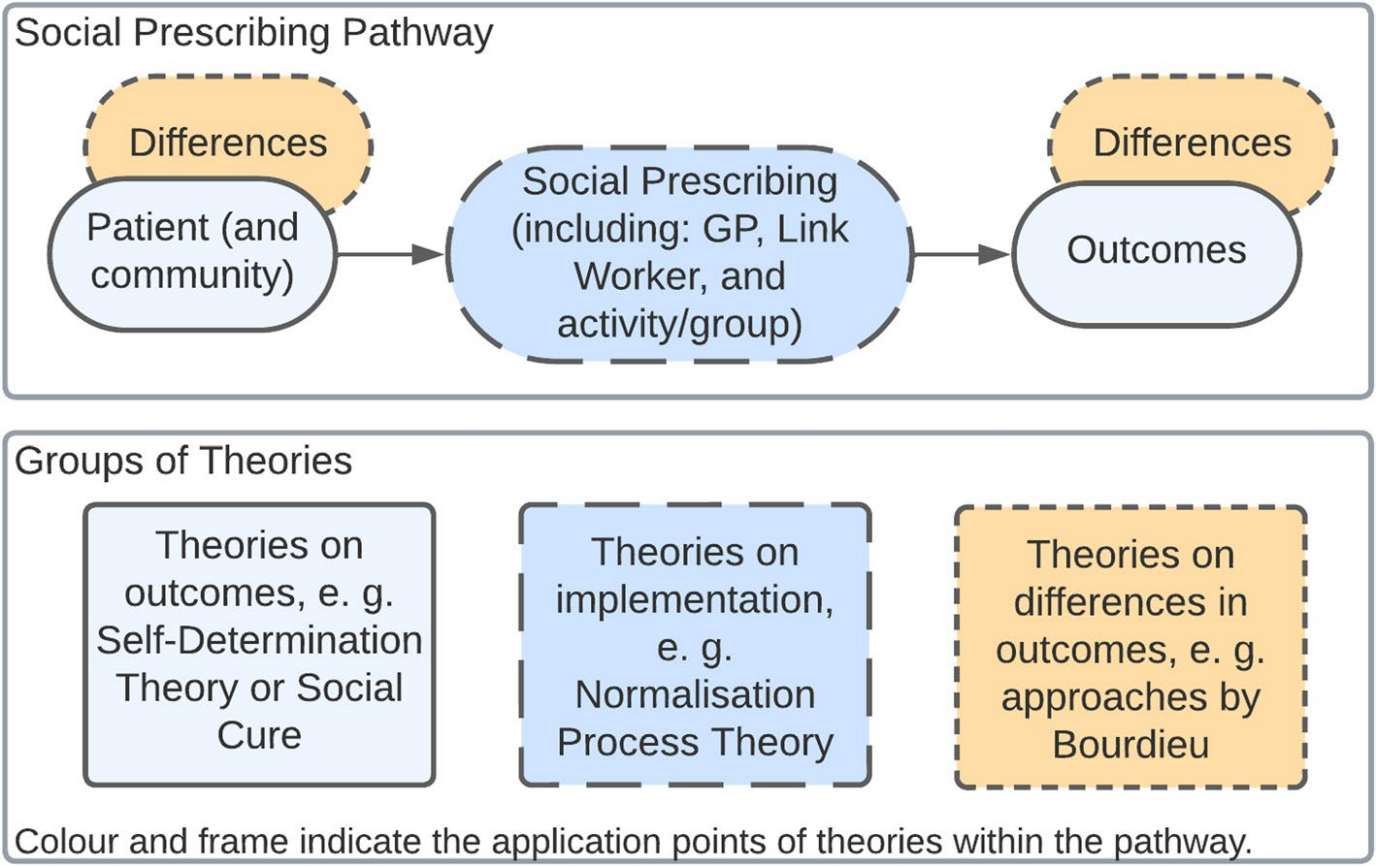
The use of theories for social prescribing with examples

The 18 included reports employed 11 distinct theories, which we sorted into three groups according to the aspect of the SP pathway they were used for. Four theories were used to explain how SP generates outcomes (Social Cure, Salutogenesis, Social Innovation, Self-Determination Theory), three theories were used to explain differences in outcomes (synchronicity/time, Bourdieusian approaches to class, Candidacy theory), and four theories were used to explain the implementation process of SP (Normalisation Process Theory, Boundary-spanning, Critical Systems Thinking, Social Capital). Figure 2 illustrates potential applications of theories in the SP pathway and provides examples.

All reports stated that they used a theory to understand or explain SP. However, the other uses of theories that authors described varied across the reports. One study used a theory as a basis for choosing participants (‘purposive sampling’), others to generalise findings or inform data collection or data analysis. In qualitative studies theories were used to develop an interview guide or to code data, while in quantitative studies variables and hypotheses were generated and tested. Two reports included three theories that we sorted into different groups. None of the reports described that a theory was used to develop the intervention. Details on how each report used the theories can be found in Table 2.

**Table 2:** Theories and their use for social prescribing in studies

Group	Theory/theories	Report	Study Type^a^	Understand, explain	Purposive sampling	Interview guide	Coding	Generate/test variables	Generalise findings
1. Outcomes	Salutogenesis	Wood et al., 2021 [22]	QL	✓					
	Self-Determination Theory (SDT)	Bhatti et al., 2021 [23]	QL	✓			✓		
		Hanlon et al., 2021 [24]	QL	✓		✓	✓		
	Social Cure	Dingle et al., 2022 [25]	P;QT	✓				✓	
		Halder et al., 2021 [26]	P;MM	✓				✓	
		Kellezi et al., 2019 [27]	MM	✓				✓	
		Wakefield et al., 2022 [28]	QT	✓				✓	
	Social Innovation	Dayson, 2017 [29]	MM	✓				✓	✓
2. Differences in Outcomes	Bourdieusian approaches to class	Gibson et al., 2022 [30]	QL	✓					
	Synchronicity; time	Gibson et al., 2021 [31]	QL	✓					
	Candidacy	*see below Mercer *et al	-	-	-	-	-	-	-
3. Implementation	Boundary-spanning	Baker & Irving, 2016 [32]	QL	✓					
	Critical Systems Thinking (CST)	Fixsen et al., 2020 [33]	QL	✓					
	Normalisation Process Theory (NPT)	Blickem et al., 2014 [34]	QL	✓			✓		
		Chng et al., 2021^b^ [35]	QL	✓			✓		
		Whitelaw et al., 2017 [36]	QL	✓	✓				
	Social Capital	White et al., 2017 [37]	QL	✓					
Several groups	NPT, SDT, Candidacy	Mercer et al., 2017a^b^ [38]	P;MM	✓		✓	✓		✓
		Mercer et al., 2017b^b^ [39]	MM	✓		✓	✓		

^a^Study types: *P* protocol, *QL* qualitative, *QT* quantitative, *MM* mixed methods

^b^Three reports for the same study: one protocol and two publications with results

The majority of reports referred to the theories throughout the whole publication. However, four reports did not include a connection to their theory in the results section of their publication [32, 33, 36, 37].

In the following we will describe each theory and how the study authors presented their use, categorised by groups.

#### Theories that are used to explain how SP generates outcomes

Theories in the first group have been used to explain outcomes related to SP, usually focussing on positive outcomes for patients or the community.

The theory of *Salutogenesis* focuses on the question of the origins of health rather than the origins of disease. Health and illness can be seen as two poles of a continuum [40, 41]. A key element is the sense of coherence that can be defined as the extent to which one has an enduring yet dynamic feeling of confidence on three aspects: (1) that the stimuli deriving from one’s internal and external environments in the course of living are structured and predictable; (2) that the internal and external resources are available to meet the demands posed by these stimuli; (3) that these demands are challenges worthy of engagement [40]. Successfully coping with stressors strengthens the sense of coherence and helps a person to move along the continuum towards health [40]. In the context of SP, Salutogenesis was used as an explanation of health behaviours at an individual level. Salutogenesis was used in one realist analysis for the formation of a programme theory. From a Salutogenic perspective, not responding to health information from professionals was not seen as an individual failure but as a failure to provide understandable information. One of the study’s findings was ‘that SP facilitated a change in perceptions of personal assets through personal and social development’, meaning that participants became more aware of what assets were available to them and more able to access them [22].

*Self-Determination Theory* (SDT) is a psychological theory of motivation, explaining how the satisfaction of certain psychological needs creates conditions that lead to self-motivation. When applied to health, SDT focusses on the process by which individuals gain motivation to initiate and sustain new behaviours. The aim is intrinsic motivation based on interest, enjoyment and satisfaction [42, 43]. In the context of SP it was used to explain how the intervention created positive outcomes for participants [23] or the impact the intervention had on them, including a change or lack of change [24]. The two qualitative studies using SDT both conducted interviews with patients, one of them additionally having focus groups [23, 24]. One conducted its qualitative analysis in two stages: The first stage was used to choose a theory that was the basis for the analysis in the second stage. The chosen theory was SDT which then informed some of the initial codes that were generated in a deductive approach [23]. The second study based its interviews on a topic guide informed by SDT and considered SDT in its coding frame for the analysis [24]. In one protocol applying a mixed methods approach, SDT was intended to assess the effectiveness of the intervention by basing the coding framework for interviews on it [38]. In the corresponding grey literature evaluation, the topic guide for interviews with patients was guided by SDT [39]. One of the findings of both qualitative studies using SDT was that within the SP intervention it was important to provide the opportunity for participants to create and deliver programs themselves. The option for volunteering was described as an enabler for empowerment through the four SDT components: autonomy, competence, relatedness, and beneficence [23, 24].

The *Social Cure* theory explains how social group memberships (e.g. family or leisure group) influence a person’s well-being and health. If a person has a sense of shared identification with one or several groups, that can support their positive self-identification. Further, groups can be a source of social support, especially under conditions of stress or illness [44]. In the context of SP, the theory was used by four studies to explain how SP can lead to positive outcomes regarding health and well-being by supporting membership in social groups [25–28]. Out of these, two were protocols and two presented results. They used the Social Cure to generate and test variables/hypotheses and as a basis for their measuring instruments. All four studies proposed measures regarding group membership/identification, loneliness, quality of life/well-being, and demographics. The measures for group membership or identification varied and included, for example, number of group memberships, group compatibility, community belonging, social support, and others. For loneliness, all four proposed the UCLA Loneliness Scale with 8 items (ULS-8). Two cited the EQ-5D for data on the health-related quality of life [26, 28] and one named the WEMWBS-14 for well-being [25]. Additionally, three studies specified measures for health service use [25–27]. These studies were the only ones that specified all their instruments based on a theory.

*Social Innovation* is proposed to be an adequate concept to understand and create social change. It is defined as ‘a novel solution to a social problem that is more effective, efficient, sustainable, or just than existing solutions’. Other innovations may also target social problems or aim to fulfil social needs. Social Innovation distinguishes itself by prioritizing the broader benefit to society. The objective is to generate social value that is defined as creation of benefits or reduction of costs for society, i.e. beyond private gains [45]. Therefore, Social Innovation and social value are closely linked. In one SP study the concept of Social Innovation informed the evaluation and is used to operationalise and measure the value generated by the intervention. The aim of the mixed methods multi-stakeholder approach was to identify the different types of social value and to generalise the approach of measuring value for other interventions. The study defined three types of social value: hospital resources, well-being, and wider/unintended values. To assess the first, hospital data was included. For the other two types, a well-being questionnaire for patients was used as well as interviews with patients, their caregivers, commissioners, and providers. Using social value in this study emphasised the benefits of including unintended social value in an evaluation. The study described the process of acquiring funding for the SP service and the important role, the qualitative data played in displaying the breadth of value that was created by the intervention. It concluded that there were advantages of a mixed methods approach to evaluate SP [29].

#### Theories that are used to explain differences in outcomes

Theories in the second group are used to explain why different (groups of) participants have differing outcomes. The three included theories are used to explain how inequalities shape access and possibilities of participation.

*Bourdieu’s* theoretical concepts include class, habitus, and capital. Habitus is the internalised system by which someone generates meaningful practices and meaning-giving perceptions. It encompasses both, our behaviour and our perceptions of the behaviours of those around us. Capital exists in three forms: economic, social and cultural [46]. In the context of SP, the theory was used to describe how class and classed habitus influence a participant’s engagement in the intervention through accumulation of (social) capital. An ethnographic study included four case studies of participants and assessed how class enabled or constrained their participation [31]. For example, a harmonious transition back into a gym was interpreted as based on inherited health capital, because the participant has been using the gym at a younger age. Another participant’s social network acted as a form of social capital, because it supported them to access opportunities that the intervention offered. Challenging immediate social circumstances served as an example of how external factors limit individuals’ choices. For instance, one individual had a history of trauma along with difficult circumstances at the moment of the intervention and therefore limited inherited capital to exchange for the provided health opportunities.

The authors illustrate with this example how non-attendance at appointments does not necessarily indicate a lack of motivation. In the context of poverty, individuals may resort to reactive strategies aimed at acquiring economic capital rather than investing in their future health. The theory was used to understand health inequalities and to evaluate the intervention [31].

The concept of asynchronicity is portrayed with regard to *time* and *synchronicity*. Individuals perceive time by differentiating into before/after. Temporal ordering happens by sequencing. The concept of asynchronicity indicates that globalising and increasingly virtual societies accelerate the intersection of time and space, i.e. simultaneity. Abilities of dealing with simultaneity increase but remain limited [47]. In the context of SP, the concepts of time and synchronicity were used to contrast the experience of time by participants as complex/changeable and that of an intervention that assumes a linear and constant trajectory of health and wellbeing. For example, in one case study a participant was waiting for information on a court case of her child. Asking questions about her immediate health status is described as seeming ‘mundane’ in contrast to what is happening at the same time. The aim of the study was to ‘understand where and why services fail to deliver timely care’ [30].

*Candidacy* is used in the context of healthcare access for vulnerable groups. It considers how ‘eligibility for medical attention and intervention is jointly negotiated between individuals and health services’ and how access requires work on the part of the users [48]. In the context of SP, it is meant to, ‘explain[...] the process through which people see themselves as ‘candidates’ for a referral or how a professional decides whether to refer a patient’ [38]. The authors described the planned use in their protocol [38] and explored the usefulness of candidacy theory by incorporating its constructs into their coding framework [39]. However, in their evaluation they concluded that the theory was not useful to them and therefore did not apply it for the analysis of their results [39].

#### Theories that are used to explain the implementation process

These theories were used to explain the process of the implementation of SP, often concerning different professions working together.

*Boundary-spanners* are described as individuals with specific skills, competencies, and behaviour who work across sectoral and organisational boundaries, which is necessary to address complex societal problems [49]. In the context of SP, this concept was used to explain collaboration between different professions. The authors of the interview study stated that the concept of Boundary-spanners was used to classify and analyse Boundary-spanning activity. They did not provide further clarification on what that means. One of their findings was that GPs and social professionals operated within different institutional logics, for example having a different understanding of health. To establish a working co-production between different professions in an intervention they described the need for a shared understanding, for example about the purpose of the program [32].

*Critical Systems Thinking* (CST) seeks to consider complexity to analyse contexts as well as constructing interventions. CST is theoretically based on social theory and meant to be practically applicable by additionally integrating systems thinking. The result is a variety of methods [50]. CST was named in one study but barely described. The authors stated that it was used to understand how SP is implemented and to examine ‘the vision, aspirations and boundary judgments of a local social prescribing scheme’. Other than that, they named several questions they based on the theory, for example: ‘What boundary judgements were made and why?’ [33].

*Normalisation Process Theory* (NPT) provides a set of tools to understand how practices become routinely embedded in everyday life, recognising the social, psychological, organisational and societal factors that influence the adoption of new practices. It includes, for example, experiences and beliefs as well as availability of resources [51]. Five reports applied NPT [34–36, 38, 39]. Three of them were qualitative studies. One used NPT to purposively sample general practices based on their willingness to be involved [36] and two used it for coding their interview data [34, 35]. One of them closely linked its results section to the theory by using the components of NPT to sort general practices into fully and partially integrated practices according to NPT variables [35]. In the protocol for this study, that is also included in this review, they planned to base their interview and coding guideline on NPT [38]. In their grey literature evaluation they further described that their interviews were guided by NPT and that the codes were mapped according to NPT [39]. One finding from using NPT was that only three of seven practices fully implemented the programme as planned, even though it was a well-funded government programme over a 2-year period. While the fully and partially integrated groups did not differ in list size or ethnicity of patients, successful implementation was, for example, influenced by leadership and team relationships [35].

Theories on *Social Capital* informed a framework that describes a model for improving relationships within primary care practices ‘to promote organizational success and improve patient care outcomes’. This framework comprises the three theoretical dimensions of structural (bonding, bridging), relational (cooperating, trusting) and cognitive (shared understanding) Social Capital [52]. In the context of SP, this framework based on theories was used to explain the relationships between different professions. As an example, a result of this study was that a mutual understanding is an important basis for successfully working together as well as trust. In their SP intervention, they identified mistrust by primary care physicians towards third sector organisations as a difficulty [37].

## Discussion

The aim of this scoping review was to describe theories that are used for SP to better understand how implementation or evaluation might differ based on different theoretical approaches. We located 11 theories that we categorised into three groups: outcomes, differences in outcomes, and implementation of SP.

### Using theories to define outcome measures

We grouped Salutogenesis, SDT, the Social Cure and Social Innovation as theories explaining the outcomes of SP. The first three of these were used by studies to explain individual-level improvements in health [22–28]. As an example, in one study the Social Cure theory provided a causal pathway implying that SP leads to a higher number of group memberships, which leads to a higher level of community belonging, leading to less loneliness, and in turn less primary care use [27]. This potentially explains how changes in the number of group memberships can result in less primary care use.

In contrast, Social Innovation was used to explicate impacts at a broader (i.e. societal) level, with authors emphasising the need to monitor unexpected or unintended consequences of SP. One unexpected positive outcome was that voluntary and community organisations were able to leverage additional funding through their involvement in SP programmes. The research team suggested that avoiding narrow definitions of impacts around SP allowed these important findings to be captured and recommended a mixed methods approach for evaluation [29].

The two studies described above demonstrate that using different theoretical approaches enables identifying different outcomes but also various methods of assessing them. The first theory was used to define variables and test their causal relationships, while the second theory guided the recommendation to use mixed methods when evaluating SP.

### Using theories to consider social inequalities

In contrast to the first group, the three theories in the second group specifically focused on differences in outcomes: Candidacy, time/synchronicity and Bourdieusian approaches to class. One of them, Candidacy, is only briefly mentioned in a study protocol but has not been further applied [35, 38, 39]. The other two theories were used in one study each. Both studies were written by the same group of authors, focused on individual cases, and shared a similar line of argument. Both studies emphasised how SP can potentially perpetuate inequalities and stressed the importance of considering contextual factors and accommodate different needs when developing and implementing SP. Additionally, they questioned if an individual-level intervention can address the determinants of health and structural disadvantages, emphasising the need to address inequalities not only with SP [30, 31].

Similarly, Brown et al. had emphasised the importance of social justice and highlighted the inverse care law to underscore the need for SP to consider existing social inequalities when implementing SP [53]. They warned that claims made around the role of SP in addressing inequalities could be misused by policy makers to create an impression of addressing health inequalities without adequately fulfilling people’s broader social and economic needs, if SP is seen as a ‘cheaper and less disruptive’ alternative to e. g. addressing poverty [53].

The themes addressed by the theories in this group are central to SP, which aims at decreasing social inequalities. However, Candidacy was initially intended for use in the protocol but was considered unhelpful in the actual evaluation. The other two theories are characterised by their broad and abstract nature, potentially posing challenges in practical application.

There are a few overlaps between our first and second group of theories: Some theories used to explain outcomes are used to *additionally* explain ‘differences in outcomes’. For example the Social Cure was used to state that group participation only creates positive outcomes if an individual identifies with that group [25–28]. Another team argued that using SDT as a psychological theory of individual change does not fully reflect circumstances [24].

### Using theories for the planning of SP interventions

Surprisingly, we encountered no study describing the development of theory-based interventions. Instead, all theories were employed for the evaluation of services. Consequently, the precise ways in which theories contribute to the design of SP interventions remain unclear. This includes, for example, a theory’s influence on aspects such as the target group, intervention objectives, or strategies for enabling change. If theories play a guiding role in intervention design, it would be of value to specify how they were applied.

The theories we would particularly expect to be used in the planning process of the intervention are theories on the implementation process. In contrast to the other groups, the four theories we found on the implementation were not necessarily restricted to topics around health or well-being. Instead, they focused on aspects such as interprofessional collaboration and emphasised questions such as:

- How well did the communication between different stakeholders work [32]?
- What shared experiences or concerns did the different stakeholders have [33]?
- How was successful implementation influenced by leadership and team dynamics [35]?

The included studies reporting these theories noted the importance of considering these aspects, in line with prior work on primary care highlighting ideological, organisational, and structural challenges impacting on interprofessional collaboration [54]. Importantly, these barriers were noted in one study as impacting on the uptake of SP, with around half of participating general practices only partially implementing the SP intervention [35].

The implementation of SP is highly challenging, as it often not only requires structural adjustments but also a shift in attitudes across various professions. Proactive planning and thoughtful contemplation of strategies are essential for effectively addressing the challenges associated with interprofessional and intersectoral collaboration. Employing a theory to guide the implementation process can mitigate the risk of overlooking these challenges.

### Challenges in theory application

The three groups illustrate the diversity of the theories, each addressing distinct aspects. In addition, the theories within these groups enable varied applications and emphasise different focal points, resulting in differing outcomes. This prompts the question of whether employing a single theory is adequate to encompass the relevant mechanisms of a SP intervention. In fact, one study intended to incorporate three theories for comprehensive coverage: This study was described in three reports [35, 38, 39]. In the protocol the authors aimed to use the RE-AIM framework and proposed three different theories (NPT, SDT, and Candidacy theory) for different dimensions of the framework [38]. We classified NPT, SDT and Candidacy into three different groups for this review. However, the corresponding journal article only described the use of one of the proposed theories (NPT) [35]. In their evaluation, the authors described only NPT as useful [39]. In contrast, two other studies used SDT and described its components as fitting for SP [23, 24]. The different perceptions on SDT being a helpful theory for the evaluation possibly illustrates the complexity of theory-adoption in SP.

The majority of the included reports applied theories for coding qualitative data or interpreting results. Only the Social Cure theory was used in a quantified way: it provided a theoretical framework for selecting outcomes/measures in a quantitative approach, enabling the generation and testing of hypotheses [25–28]. The fact that only one team of authors undertook this approach may suggest the challenges in operationalising theories for quantitative methods.

A reason for theories not being used for planning SP can be a perceived gap between theory and practice. Similar findings were described for public health practitioners who indicate difficulties with and concerns about using behavioural sciences to design interventions [55].

The studies included in the results of this review suggest that the application of theory presents challenges. However, some studies used theories and reported them as beneficial.

### Gaps in the literature

We observed two major gaps in the literature. Firstly, most studies do not provide clear information on whether and which theory was employed. Secondly, even in studies that do mention the use of a theory, there is often a lack of comprehensive details on how the theory was applied and whether it was perceived as appropriate and value-adding.

Of the 18 reports that explicitly incorporated theory, 14 consistently referred to these theories throughout the publication. The remaining four reports detailed a theory and how it was used, but did not draw a connection to their results section [32, 33, 36, 37]. Two of the 18 reports described how they used a theory but did so rather brief and vaguely [22, 32]. In these studies, it was difficult to understand the actual role and the connection between the theory and the results [22, 32, 33, 36, 37]. In some studies, the results appeared detached from the theory and the description of theory use was adding little value for the reader.

Having access to comprehensive information on theory use is essential for understanding what makes a theory useful for SP and how to employ it effectively. Unfortunately, there is a notable scarcity of such information, which may be a contributing factor to the limited utilisation of theories in this field. It should be noted that only very few studies were found in this literature review that described the use of theories at all. This shortcoming is mainly due to the fact that the interventions were not developed on the basis of theory. However, for an evidence-based intervention and for a methodologically valid evaluation, theories have to be considered at the beginning when designing the intervention.

### Strengths and limitations

This study represents the first systematic effort to provide an overview of the theories used and how they were applied in the context of SP. We conducted an extensive literature search, including nine databases, contacting authors by email, and using Twitter. The strength of this study lies in its broad coverage of the topic, giving a comprehensive overview, and identifying gaps in the existing literature, thus guiding future research directions.

To ensure consistency and focus on the most common adoption of SP globally, we included only interventions that involved a professional with a linking function to various support sources. It is worth noting that other models may employ different theories.

For the inclusion criteria we had to define ‘theory’. It was challenging to decide whether synchronicity/time [30], Boundary-spanning [32], and Social Innovation [29] fulfilled the criteria. We included all three studies due to the broad scope we aimed to cover.

As a limitation, this study only incorporated publications in the English language. Furthermore, our reliance on information provided by the authors in their publications may not have been comprehensive due to word limits imposed by journals. We did not seek additional clarification from the authors.

### Implications of the findings for research and practice

Theories offer explanations for how interventions work, thereby providing guidance for the development and evaluation. Each of the groups that theories were sorted into encompasses relevant aspects for SP interventions. The first group addresses how SP is intended to function and who is meant to benefit (e. g. individuals whose health or well-being can be enhanced through belonging to a social group or by them changing their behaviour). The second group expands on this by adding a focus on the context of individual circumstances (e. g. trauma or poverty), with the notion that SP cannot by itself address social inequalities. The third group focuses on the foundational aspect that in order to achieve positive outcomes with SP, it must be implemented first (e. g. ensuring that the workforce fully understands what SP is, its objectives, and operational procedures). Nevertheless, a description of the use of a theory was only found in 18 reports, in some of them only in a few sentences. This may suggest an inadequate description of theory use or a lack of use.

An outstanding research question is, for which reasons theories are not employed in the planning or evaluation of SP interventions. Furthermore, future investigations should explore the efficacy of different theories in specific contexts. Additionally, there is a need to examine practical methods for applying and operationalising theories to improve service delivery. Lastly, determining the most appropriate qualitative, quantitative, or mixed method approaches for evaluating interventions when using theories should be an area of interest. Gaining insight into these aspects can enhance our understanding of who can use which theory (or combination of theories) and in what ways, in order to effectively enhance SP interventions.

## Conclusion

This scoping review aimed to provide an overview of how theories were used in the development or evaluation of SP. We did not identify any study that described using a theory to develop an intervention. Only few studies described how they used a theory for their evaluation. Either the majority of studies were not led by theory or it was not described in the publications. It appears crucial to include information in publications regarding whether and how a theory was employed, along with its appropriateness, in order to expand the knowledge base. Among the descriptions of the included studies, various theories were employed either singularly or in combination to comprehend SP and guide data collection and analysis. These theories influenced outcome measures, the presumed causal pathway, and methodological approaches. The studies involved 11 distinct theories, which were categorised into three groups: outcomes (e.g., the Social Cure), differences in outcomes (e.g., Bourdieusian approaches to class), and implementation (e.g., Normalisation Process Theory). Notably, all three groups of theories encompass relevant aspects for SP interventions, addressing questions aligned with three main goals of SP: successful implementation, fostering positive patient/community outcomes, and addressing inequalities. Further efforts are needed to explore practical applications and operational methodologies for effectively integrating theories into enhancing SP interventions.

### Supplementary Information

**Additional file 1: Supplementary file 1.** Search strategy for the database search.

**Additional file 2: Supplementary file 2.** Additional literature search.

**Additional file 3: Supplementary file 3.** Data extraction instrument.

## Data Availability

The dataset supporting the conclusions of this article is included within the article and its additional files.

## Abbreviations

CST: Critical Systems Thinking
GP: General practitioner
NPT: Normalisation Process Theory
SDT: Self-Determination Theory
SP: Social prescribing
