# Supplementary material for: Theories used to develop or evaluate social prescribing in studies: a scoping review
Source: BMC Health Serv Res. 2024 Jan 26;24:140. doi: 10.1186/s12913-024-10563-6 (PMC10821232; doi:10.1186/s12913-024-10563-6)
Supplement: Supplementary file 1 — Additional file 1: Supplementary file 1. Search strategy for the database search. [file 12913_2024_10563_MOESM1_ESM.docx]

# **Supplementary File 1: Search Strategy for the database search**

## Table s1: Pilot search: PubMed search string and number of results (conducted 08.07.2022)

|  | Search | No. results |
| --- | --- | --- |
| #1 | “social prescri*”[Tiab] | 312 |
| #2 | “social referral*”[Tiab] | 14 |
| #3 | “community prescri*”[Tiab] | 111 |
| #4 | “community referral*”[Tiab] | 204 |
| #5 | “community-based prescri*”[Tiab] | 7 |
| #6 | “community-based referral*”[Tiab] | 36 |
| #7 | “link worker*”[Tiab] | 101 |
| #8 | “community connector*”[Tiab] | 17 |
| #9 | #1 OR #2 OR #3 OR #4 OR #5 OR #6 OR #7 OR #8 | 738 |

## Table s2: Database Search with search strings and number of results (conducted 19.07.2022)

| Database | Search String | No. results |
| --- | --- | --- |
| Pubmed | “social prescri*”[Tiab] OR „social referral*"[Tiab] OR „community referral*"[Tiab] OR „community prescri*"[Tiab] OR „community-based prescri*"[Tiab] OR „community-based referral*"[Tiab] OR "link worker*"[Tiab] OR "community connector*"[Tiab] | 740 |
| CINAHL | TI ( "social prescri*" OR "social referral*" OR "community referral*" OR "community prescri*" OR "community-based prescri*" OR "community-based referral*" OR "link worker*" OR "community connector*" ) OR AB ( "social prescri*" OR "social referral*" OR "community referral*" OR "community prescri*" OR "community-based prescri*" OR "community-based referral*" OR "link worker*" OR "community connector*" ) | 545 |
| ASSIA | ti(“social prescri*” OR „social referral*" OR „community referral*" OR „community prescri*" OR „community-based prescri*" OR „community-based referral*" OR "link worker*" OR "community connector*") OR ab(“social prescri*” OR „social referral*" OR „community referral*" OR „community prescri*" OR „community-based prescri*" OR „community-based referral*" OR "link worker*" OR "community connector*") | 181 |
| Cochrane | “Social prescribing” OR “Social prescription” OR “Social referral” OR “Community referral” OR “Community prescribing” OR “Community prescription” OR "Community-based referral" OR “Link worker” OR “Community connector” in Title Abstract Keyword | 119 |
| PsycINFO | (Social prescribing OR Social prescription OR Social referral OR Community referral OR Community prescribing OR Community prescription OR Community-based referral OR Link worker OR Community connector).ti. or (Social prescribing OR Social prescription OR Social referral OR Community referral OR Community prescribing OR Community prescription OR Community-based referral OR Link worker OR Community connector).ab. | 177 |
| Web of Science | TI=("social prescri*") OR TI=("social referral*") OR TI=("community referral*") OR TI=("community prescri*") OR TI=("community-based prescri*") OR TI=("community-based referral*") OR TI=("link worker*") OR TI=("community connector*") OR AB=("social prescri*") OR AB=("social referral*") OR AB=("community referral*") OR AB=("community prescri*") OR AB=("community-based prescri*") OR AB=("community-based referral*") OR AB=("link worker*") OR AB=("community connector*") | 825 |
| Scopus | TITLE-ABS-KEY ( "social prescri*" OR "social referral*" OR "community referral*" OR "community prescri*" OR "community-based prescri*" OR "community-based referral*" OR "link worker*" OR "community connector*" ) | 1049 |
| Sociological Abstracts | ti(“social prescri*” OR „social referral*" OR „community referral*" OR „community prescri*" OR „community-based prescri*" OR „community-based referral*" OR "link worker*" OR "community connector*") OR ab(“social prescri*” OR „social referral*" OR „community referral*" OR „community prescri*" OR „community-based prescri*" OR „community-based referral*" OR "link worker*" OR "community connector*") | 52 |
| Social Care Online | (Title (“social prescribing” OR “social prescription” OR “community referral” OR “community prescribing” OR “community prescription” OR “community-based prescription” OR “community-based referral” OR “link worker” OR “community connector”)) OR (Abstract (“social prescribing” OR “social prescription” OR “social referral” OR “community prescription” OR “community-based prescription” OR “community-based referral” OR “link worker” OR “community connector”)) | 284 |
