# Supplementary material for: Theories used to develop or evaluate social prescribing in studies: a scoping review
Source: BMC Health Serv Res. 2024 Jan 26;24:140. doi: 10.1186/s12913-024-10563-6 (PMC10821232; doi:10.1186/s12913-024-10563-6)
Supplement: Supplementary file 2 — Additional file 2: Supplementary file 2. Additional literature search. [file 12913_2024_10563_MOESM2_ESM.docx]

# **Supplementary file 2: Additional literature search**

## Table s1: Text for Email for contacting experts

| Theory in social prescribing - Call for evidence  Hi …,  For a scoping review we are collating studies that use theory for developing or evaluating social prescribing. The protocol for this review can be found here: https://osf.io/k9tgx/ .  In addition to our database and grey searches, and given your experience in the area, we wondered if you would mind forwarding any thoughts, links to studies we should be aware of, or others we should contact please?  Thank you, hugely appreciated in advance,  Sinah Evers |
| --- |
| Hi All,  In a project led by Sinah Evers at the University of Bremen, we are collating studies that use theory for developing or evaluating social prescribing. The protocol for this review can be found here: https://osf.io/k9tgx/ .  In addition to our database and grey searches, and given you’ve all such experience in the area, we wondered if you would mind forwarding any thoughts, links to studies we should be aware of, or others we should contact please? Feel free to reply here or contact Sinah at: sinah.evers@uni-bremen.de  Thank you, hugely appreciated  Kerryn Husk |

## Figure s1: Twitter call


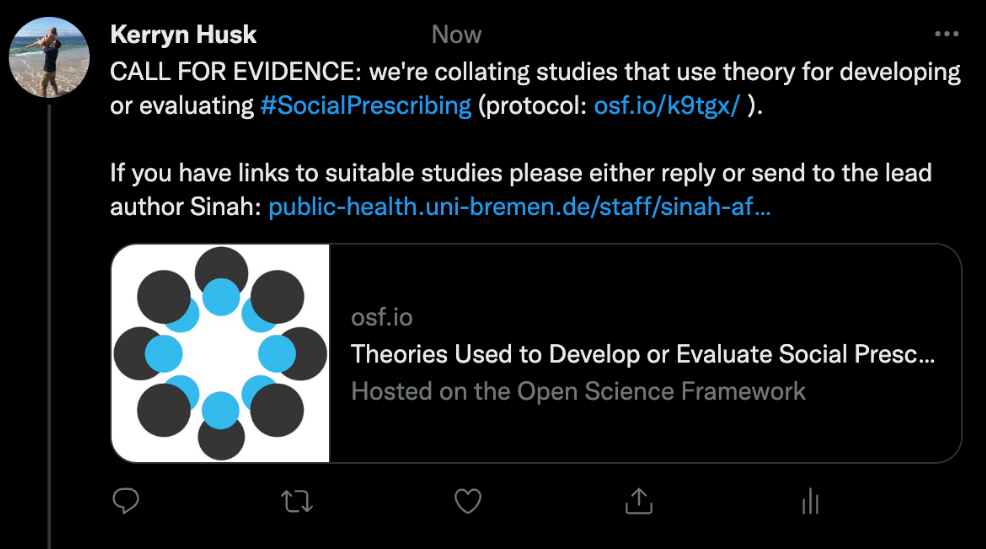


## Table s2: List of results from expert contact and twitter call

| Origin | Source |
| --- | --- |
| Twitter | Sandhu, S., Lian, T., Drake, C., Moffatt, S., Wildman, J., & Wildman, J. (2022). Intervention components of link worker social prescribing programmes: A scoping review. Health & Social Care in the Community, 00, 1–14. https://doi.org/10.1111/hsc.14056 |
| Twitter | Bhatti S, Rayner J, Pinto AD, Mulligan K, Cole DC. Using self-determination theory to understand the social prescribing process: a qualitative study. BJGP Open. 2021 Apr 26;5(2):BJGPO.2020.0153. doi: 10.3399/BJGPO.2020.0153. PMID: 33402331; PMCID: PMC8170608 |
| Expert | Bertotti M, Frostick C, Hutt P, Sohanpal R, Carnes D. A realist evaluation of social prescribing: an exploration into the context and mechanisms underpinning a pathway linking primary care with the voluntary sector. Prim Health Care Res Dev. 2018 May;19(3):232-245. doi: 10.1017/S1463423617000706. Epub 2017 Dec 7. PMID: 29215328; PMCID: PMC5904290. |
| Expert | Fixsen, A., Seers, H., Polley, M. *et al.* Applying critical systems thinking to social prescribing: a relational model of stakeholder “buy-in”. *BMC Health Serv Res* **20**, 580 (2020). https://doi.org/10.1186/s12913-020-05443-8 |
| Expert | Gemma Bradley & Jason Scott (2021) Social Prescribing Nomenclature, Occupational Therapy and the Theory of Institutional Work: Creating, Maintaining and Disrupting Medical Dominance, Occupational Therapy In Health Care, DOI: 10.1080/07380577.2021.1926046 |
| Expert | Chng NR, Hawkins K, Fitzpatrick B, O'Donnell CA, Mackenzie M, Wyke S, Mercer SW. Implementing social prescribing in primary care in areas of high socioeconomic deprivation: process evaluation of the 'Deep End' community Links Worker Programme. Br J Gen Pract. 2021 Nov 25;71(713):e912-e920. doi: 10.3399/BJGP.2020.1153. PMID: 34019479; PMCID: PMC8463130. |
| Expert | Dayson, C. 2017. Policy commentary - Social prescribing “plus”: a model of asset-based collaborative innovation? People, Place and Policy Online 11(2), pp. 90–104. doi: 10.3351/ppp.2017.4839587343. |
| Expert | Bartels, Davies-McIntosh, Howard, Loeffler, Powell, Pykett, Shaw & Woodcock (2022). Social Prescribing, Assets and Relationships in Communities (SPARC): Co-producing a Social  Model of Wellbeing  Position paper at: https://www.birmingham.ac.uk/research/centre-urban-wellbeing/themes/social-prescribing-assets-and-relationships-in-communities-sparc-network.aspx |
| Expert | Sandhu S, Lian T, Drake C, Moffatt S, Wildman J, Wildman J. Intervention components of link worker social prescribing programmes: A scoping review. Health Soc Care Community. 2022 Oct 1. doi: 10.1111/hsc.14056. Epub ahead of print. PMID: 36181384. |
| Expert | Bhatti S, Rayner J, Pinto AD, Mulligan K, Cole DC. Using self-determination theory to understand the social prescribing process: a qualitative study. BJGP Open. 2021 Apr 26;5(2):BJGPO.2020.0153. doi: 10.3399/BJGPO.2020.0153. PMID: 33402331; PMCID: PMC8170608. |
| Expert | Hanlon P, Gray CM, Chng NR, Mercer SW. Does Self-Determination Theory help explain the impact of social prescribing? A qualitative analysis of patients' experiences of the Glasgow 'Deep-End' Community Links Worker Intervention. Chronic Illn. 2021 Sep;17(3):173-188. doi: 10.1177/1742395319845427. Epub 2019 May 3. PMID: 31053038. |
| Expert | Aughterson, H., Baxter, L. & Fancourt, D. Social prescribing for individuals with mental health problems: a qualitative study of barriers and enablers experienced by general practitioners. *BMC Fam Pract* **21**, 194 (2020). https://doi.org/10.1186/s12875-020-01264-0 |
| Expert | Hayes D, Edbrooke-Childs J, Town R, Wolpert M, Midgley N. Barriers and facilitators to shared decision making in child and youth mental health: clinician perspectives using the Theoretical Domains Framework. Eur Child Adolesc Psychiatry. 2019 May;28(5):655-666. doi: 10.1007/s00787-018-1230-0. Epub 2018 Sep 18. PMID: 30229306; PMCID: PMC6514112. |
| Expert | Fixsen, A., Seers, H., Polley, M. et al. Applying critical systems thinking to social prescribing: a relational model of stakeholder “buy-in”. BMC Health Serv Res 20, 580 (2020). https://doi.org/10.1186/s12913-020-05443-8 |
| Expert | Hamilton-West K, Gadsby E, Zaremba N, Jaswal S. Evaluability assessments as an approach to examining social prescribing. Health Soc Care Community. 2019 Jul;27(4):1085-1094. doi: 10.1111/hsc.12726. Epub 2019 Feb 5. PMID: 30723977. |
| Expert | Griffith B, Pollard T, Gibson K, Jeffries J, Moffatt S. Constituting link working through choice and care: An ethnographic account of front-line social prescribing. Sociol Health Illn. 2022 Oct 25. doi: 10.1111/1467-9566.13569. Epub ahead of print. PMID: 36284215. |

##

## Table s3: List of searched websites

| **Organisation** | **Website** |
| --- | --- |
| Social Prescribing Network | <https://docs.google.com/spreadsheets/d/1ofgO0c0EH6SW4N7XP8gXtSFK7oTCwBLIIdG2n4BS9XY/edit#gid=340532624> |
| NHS Website | <https://www.england.nhs.uk/personalisedcare/social-prescribing/> |
| NASP Website | <https://socialprescribingacademy.org.uk/our-work/evidencing-social-prescribing/social-prescribing-the-evidence/> |
| Google Scholar | intitle:”social prescribing“ AND theory // No patents or citations |
| NHS Platform | <https://future.nhs.uk/system/login?nextURL=%2Fconnect%2Eti%2Fsocialprescribing%2Fgrouphome> |
| Alliance for Healthier Communities | <https://www.allianceon.org/Social-Prescribing> |
| CISP | <https://www.socialprescribing.ca/> |
| Google | allintitle: social prescribing theory |
|  | <https://www.ntusocialidentity.com/socialprescribing> |
