# Supplementary material for: Theories used to develop or evaluate social prescribing in studies: a scoping review
Source: BMC Health Serv Res. 2024 Jan 26;24:140. doi: 10.1186/s12913-024-10563-6 (PMC10821232; doi:10.1186/s12913-024-10563-6)
Supplement: Supplementary file 3 — Additional file 3: Supplementary file 3. Data extraction instrument. [file 12913_2024_10563_MOESM3_ESM.docx]

**Supplementary file 3: Data Extraction Instrument**

## Table s1: Data extraction on the studies

| Author(s) | Year | Title | Country | Methods | Theory | Sources of the theory | Group of theory | Planning or evaluation? |
| --- | --- | --- | --- | --- | --- | --- | --- | --- |
|  |  |  |  |  |  |  |  |  |

## Table s2: Data extraction on theory and use of theory in the study

| Description of the theory |  |
| --- | --- |
| Description of the theory based on study |  |
| Description of the use |  |
